# Research on the Extraction of Hazard Sources along High-Speed Railways from High-Resolution Remote Sensing Images Based on TE-ResUNet

**DOI:** 10.3390/s22103784

**Published:** 2022-05-16

**Authors:** Xuran Pan, Lina Yang, Xu Sun, Jingchuan Yao, Jiliang Guo

**Affiliations:** 1 College of Artificial Intelligence, Tianjin University of Science and Technology, Tianjin 300457, China; pxr@tust.edu.cn; 2 National Engineering Center for Geoinformatics, Aerospace Information Research Institute, Chinese Academy of Sciences, Beijing 100094, China; yangln@aircas.ac.cn; 3 Key Laboratory of Computational Optical Imaging Technology, Aerospace Information Research Institute, Chinese Academy of Sciences, Beijing 100094, China; 4 State Key Laboratory for High-Speed Track Technology, China Academy of Railway Sciences Corporation Limited, Beijing 100081, China; yjcwxj@126.com (J.Y.); lion.g@163.com (J.G.)

**Keywords:** high resolution remote sensing image, hazard source extraction, semantic segmentation, texture enhancement, Lovász loss function

## Abstract

There are many potential hazard sources along high-speed railways that threaten the safety of railway operation. Traditional ground search methods are failing to meet the needs of safe and efficient investigation. In order to accurately and efficiently locate hazard sources along the high-speed railway, this paper proposes a texture-enhanced ResUNet (TE-ResUNet) model for railway hazard sources extraction from high-resolution remote sensing images. According to the characteristics of hazard sources in remote sensing images, TE-ResUNet adopts texture enhancement modules to enhance the texture details of low-level features, and thus improve the extraction accuracy of boundaries and small targets. In addition, a multi-scale Lovász loss function is proposed to deal with the class imbalance problem and force the texture enhancement modules to learn better parameters. The proposed method is compared with the existing methods, namely, FCN8s, PSPNet, DeepLabv3, and AEUNet. The experimental results on the GF-2 railway hazard source dataset show that the TE-ResUNet is superior in terms of overall accuracy, F_1_-score, and recall. This indicates that the proposed TE-ResUNet can achieve accurate and effective hazard sources extraction, while ensuring high recall for small-area targets.

## 1. Introduction

**Figure 1:**
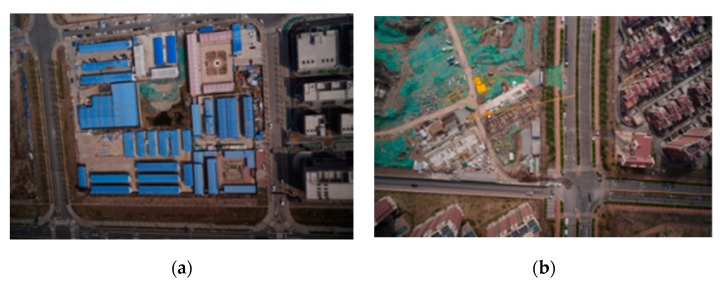
Hazard source along high-speed railways in high resolution remote sensing image: (**a**) color-coated steel house; (**b**) dust-proof net.

Hazard sources, such as aging color-coated steel houses, poorly fixed dust-proof nets, and mulch films, along a high-speed railway are prone to be blown up by the wind and fall on the high-speed rails or grids, thereby hindering the normal operation of high-speed railway, or even causing major security incident. Notifying the relevant departments and units to strengthen the hazard sources through the ground investigation can effectively prevent accidents from occurring. For a long time, the ground investigation method has been the main means to detect the hazard sources around the railway. However, the traditional ground investigation requires a lot of manpower and material resources, and is limited by the environmental conditions, resulting in low efficiency and poor investigation results [1]. High-resolution remote sensing images have the advantages of large-scale, continuous time and space, periodicity, easy acquisition, and low cost. The spatial resolution of high-resolution remote sensing images has reached the submeter level, which can clearly present the hazard sources along railway, as shown in Figure 1. Therefore, high-resolution remote sensing image earth object extraction technology can be applied to accurately extract and dynamically monitor information on hazard sources along the railway line.

High-resolution remote sensing image earth object extraction aims to assign a certain earth object or background label to each pixel of the remote sensing images [2], mainly using semantic segmentation technology [3,4,5,6,7,8]. In recent years, deep learning technology has developed rapidly. Compared with traditional machine learning algorithms, deep learning can extract high-level semantic features hidden in images, and has better performance in complex scenes. Therefore, deep learning based semantic segmentation methods open up a new way for remote sensing image earth object extraction [9,10,11,12,13,14,15,16,17].

The earliest semantic segmentation method used is the patch-based method. Saito et al. [18] used patch-based convolutional neural network (CNN) to extract roads and buildings from high-resolution images, and achieved good results on Massachusetts roads and buildings datasets [19]. Alshehhi et al. [20] proposed a single patch-based CNN architecture for the extraction of roads and buildings from high-resolution remote sensing data, and low-level features were integrated with CNN features in the post-processing stage to improve the performance. However, these patch-based methods suffer from limited receptive fields and large computational overheads, so it was soon surpassed by pixel-based methods. The pixel-based methods mostly follow an encoding–decoding paradigm. Maggiori et al. [21] proposed a baseline architecture for building extraction based on fully convolutional network (FCN) [3] combined with multi-layer perceptron, and provided a building extraction dataset which was named the Inria aerial image labeling dataset. Bischke et al. [22] used a cascaded multi-task loss to optimize the SegNet [8] architecture to take advantage of the boundary information of segmentation masks, and the performance can achieve certain improvements without any changes of the network architecture. In [23], Khalel et al. proposed a stack of U-Nets to automatically label the buildings from high aerial images, of which each U-Net can be regarded as the post-processor of the previous U-Net to improve the accuracy [4]. Chen et al. [24] proposed a CNN architecture to produce fine segmentation results of high-resolution remote sensing images. The architecture introduced the cascaded relation attention module to determine the relationship among different channels or positions, and adopted information connection and error correction capture, and fused the features of geo-object details. Diakogiannis et al. [25] proposed a reliable framework for semantic segmentation of remotely sensed data consisting of a ResUNet-based architecture and a novel loss function based on the Dice loss. Lan et al. [26] proposed a novel global context based dilated convolutional neural network for road extraction. In order to cope with the complex background, the architecture introduced residual dilated blocks to enlarge the receptive field, and performed pyramid pooling module to capture the multiscale global context features. Gao et al. [27] proposed an object-oriented deep learning framework to effectively extract both low-level and high-level features with limited training data. The framework leveraged residual networks with different depths to learn adjacent feature representations by embedding a multibranch architecture in the deep learning pipeline. Although the above methods have achieved the improvement of the extraction accuracy within a certain range, there are still issues with low precision of ground objects boundaries and small objects.

The distribution of hazard sources along the high-speed railway on the high-resolution remote sensing images is sparse, with many small targets. Meanwhile, the intra-class variation of the same hazardous sources is relatively large. This brings certain technical difficulties to the hazard sources extraction from high-resolution remote sensing images, and puts forward higher requirements for the accuracy and stability of the extraction model. In this paper, we propose a texture-enhanced ResUNet (TE-ResUNet) architecture for high-resolution remote sensing image railway hazard source extraction. The network follows UNet architecture, and introduces texture enhancement module to enhance the texture details of low-level features to improve the hazard source extraction accuracy, especially the boundary extraction accuracy. Moreover, the multi-scale Lovász loss function is proposed to improve the supervision of the texture enhancement modules, and further improve the stability of the model.

## 2. Materials and Methods

### 2.1. Data Labeling

Our sample data come from the multi-spectral remote sensing images of a GF2 satellite, acquired over Beijing, China. We used the LabelMe annotation tool to manually label hazard sources along the high-speed railway and to generate binary label images. The categories of hazard sources in this paper include color-coated steel houses and dust-proof nets.

**Figure 2:**
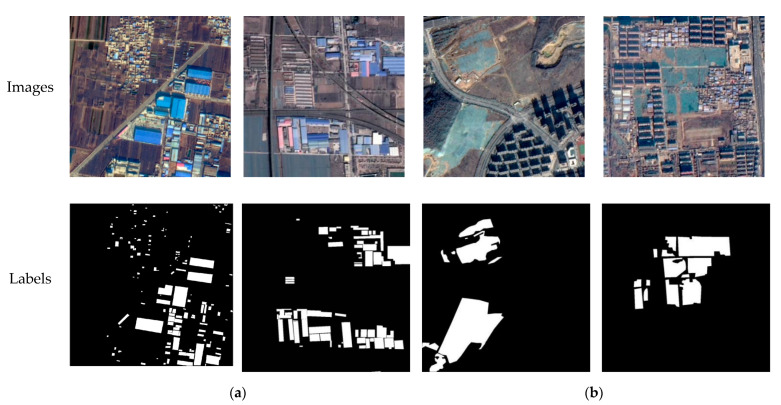
Training samples of a hazard source dataset: (**a**) color-coated steel houses; (**b**) dust-proof nets.

Color-coated steel houses are typical urban temporary buildings, with color-coated steel sheets as the raw material. The colors are mainly blue, white, and red. The color-coated steel houses in high resolution remote sensing images usually have high brightness, and the shape is mostly rectangular or rectangular combination. Dust-proof nets are mainly used for dust control and environmental protection. Exposed surfaces, dust-prone materials, etc., in cities are usually required to be covered by dust-proof nets. Dust-proof nets are generally made of polyethylene material in a light green color. Here, we define hazard source pixels as positive samples and other pixels as negative samples, and generate binary label images, as shown in Figure 2.

The original images in the hazard source dataset are the fusion images of the panchromatic and multi-spectral bands at a spatial resolution of 0.8 m, using the red, green, and blue bands. A total of 153 image patches of 1000 × 1000 pixels containing color-coated steel houses and 139 image patches containing dust-proof nets were selected for the dataset. Divided by the proportion 7:2:1 for training set, validation set, and testing set, we finally obtained 153, 43, and 21 pieces of color-coated steel house samples and 139, 39, and 19 pieces of dust-proof net samples. Data augmentation was performed on the training samples, after rotation by 90°, 180°, and 270°, horizontal and vertical inversion, and random cropping, and 3672 and 3336 image patches of 512 × 512 pixels were obtained, respectively, and the specific information is shown in Table 1.

### 2.2. Architecture of TE-ResUNet

The proposed TE-ResUNet is a segmentation network with an encoder–decoder structure based on the UNet family model-ResUNet. ResUNet is inspired by residual network, replacing each sub-module of UNet with residual connected network, which allows the network to be deepened to obtain higher-level semantic features and with a reduced risk of gradient disappearance. Typically, the UNet family models skip connect the low-level features directly to high-level features to maintain accurate texture features. However, the low-level features extracted by the shallow layer of the network are often of low quality, especially in terms of contrast, resulting in blurred texture details that negatively affect the extraction and utilization of low-level information. This, in turn, affects the extraction accuracy of the segmentation boundary. In order to make full use of the low-level texture features, a texture-enhanced ResUNet (TE-ResUnet) is proposed for fast-speed railway hazard source extraction from high-resolution remote sensing images.

**Figure 3:**
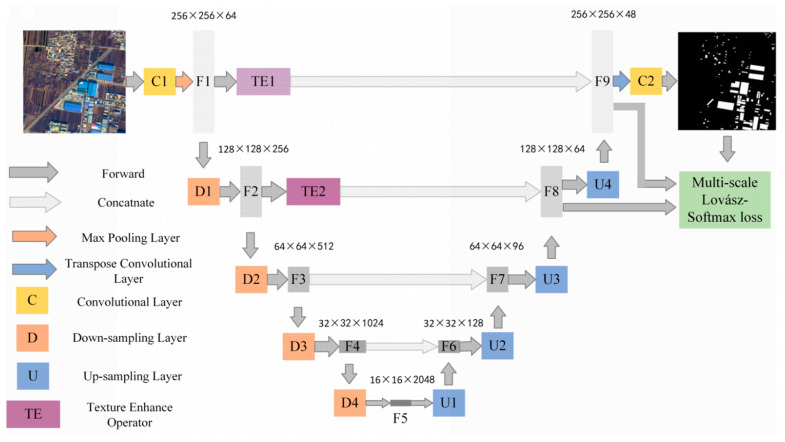
Architecture of TE-ResUNet.

The architecture of TE-ResUNet is shown in Figure 3, with a contracted path on the left side performs residual connected convolutions to produce feature maps, and an extended path on the right side to recover detailed and structural information related to hazard sources via convolution and upsampling modules. The whole network presents a U-shape. In particular, the texture enhancement modules are introduced to enhance the texture of the low-level features to assist the expansion path to better recover the hazard boundary, as well as the small targets.

The orange module D represents residual connected down-sampling module, including convolution, atrous convolution, and ReLU layers. The blue module U represents the upsampling module, including the convolution and upsampling layer, and the yellow module C represents the convolution, activation function, and batch normalization layers. The dark gray arrow represents skip connected operation. The low-level features at each scale are skip-connected to the higher layers, allowing the model to make full use of the low-level features. The low-level features extracted from the shallow layers are often of low quality, especially with low contrast, which leads to blurred texture details, and brings negative impacts on extraction and utilization of low-level information. Therefore, this paper introduces the texture enhancement module (purple module TE) to perform texture enhancement on the low-level features before they are delivered to the deeper layers by skip connections.

The texture enhancement (TE) module is inspired by histogram equalization, a classical method of image quality enhancement [28]. The TE module aims to convert histogram equalization into a learnable manner. It first encodes the feature to a learnable histogram, during which the input features are quantized into multiple level intensities, and each level can represent a kind of texture statistics. Then, a graph is built to reconstruct each gray level by propagating statistical information of all original levels for texture enhancement.

Specifically, the texture enhancement module starts by generating a histogram with the horizontal and vertical axes representing each gray level and its count value, respectively. These two axes are represented as two feature vectors, *G* and *F*. Histogram equalization aims to reconstruct the levels as *G*′ using the statistical information contained in *F*. Each level *G*′*_n_* is converted to *G*′*_n_* by Equation (1):

(1) $$G_{n}^{\prime }=\frac{\left(N-1\right)\sum_{i=0}^{n}F_{n}}{\sum_{i=0}^{N}F_{n}}$$

where *N* represents the number of gray levels.

**Figure 4:**
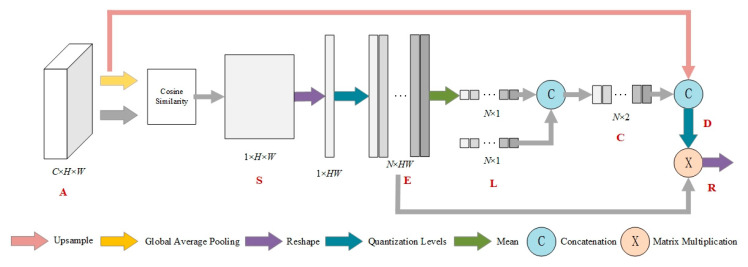
Texture enhancement module.

The structure of the texture enhancement module is shown in Figure 4. The input feature of TE module is of high channel dimensionality. In order to quantize and count the high dimensional representation, we compute the similarity between each vector and average feature as the counted object instead of quantizing each channel separately. The input feature map *A_C_*_× *H* × *W*_ is converted into the global averaged feature *g_C_*_× 1_ by global average pooling. Each spatial position *A_ij_* of *A* has dimension *C* × 1, and the cosine similarity of each *A_ij_* and the mean *g* is then calculated to obtain the similarity map *S*_1 × *H × W*_, where *S_i_*_,*j*_ can be calculated by Equation (2).

(2) $$S_{i,j}=\frac{g\cdot A_{i,j}}{{\left\|g\right\|}_{2}\cdot \|A_{i,j}\|{}_{2}}$$

*S* is then reshaped to 1 × *HW* and quantized with *N* gray levels *L*. *L* is obtained by equally dividing *N* points between the minimum and maximum values of *S*. The quantization encoding map *E_N_*_× *HW*_ is thus obtained. The histogram *C_N_*_× 2_ is represented by concatenating *E* and *L*, where *L* is the horizontal coordinate indicating the number of quantized gray levels, and *E* is the vertical coordinate indicating the weight corresponding to each level. The quantization counting map *C* reflects the relative statistics of input feature map. To further obtain absolute statistical information, the global average feature *g* is encoded into C to obtain *D*.

Afterwards, according to the histogram quantization method, a new quantization level *L*′ needs to be computed from *D*. Each new level should be obtained by perceiving the statistical information from all the original levels, and can be treated as a graph. For this purpose, a graph is constructed to propagate the information from all levels. The statistical characteristics of each quantified level are defined as a node. In a traditional histogram quantization algorithm, the adjacency matrix is a manually defined diagonal matrix, which is extended to a learning matrix as follows:

(3) $$X=Soft\;\max(\varphi_{1}\left(D{)}^{T}\cdot \varphi_{2}\left(D\right)\right)$$

where *ϕ*_1_ and *ϕ*_2_ represent two different 1 × 1 convolutional layers, and after performing Softmax operation in the first dimension as a nonlinear normalization function, each node is then updated to obtain the reconstructed quantization level ${L^{\prime }}_{C_{2}\times N}$ by fusing the features of all other nodes.

(4) $$L^{\prime }=\varphi_{3}\left(D\right)\cdot X$$

where *ϕ*_3_ represents another 1 × 1 convolutional layer.

Subsequently, the reconstruction level *L*′ is assigned to each pixel using the quantization encoding mapping *E_N_*_× *HW*_ to obtain the final output *R*, since *E* reflects the original quantization level of each pixel. *R* is obtained by:

(5) $$R=L^{\prime }\cdot E$$

*R* is then reconstructed into $R_{C_{2}\times H\times W}$, which is the final texture-enhanced feature map.

Texture enhancement of features F1 and F2 is performed by TE1 and TE2, which are then connected to the high-level features by depth to make full use of the low-level texture details to assist the network in generating more accurate hazard source extraction results. The specific model parameters of TE-ResUNet are shown in Table 2.

### 2.3. Multi-Scale Lovász Loss Function

The hazard source dataset suffers from the class-imbalance, mainly due to the sparsity of the hazard source pixel distribution. This makes the pixel-based loss function, such as cross entropy loss, focus training on background pixels that contribute less valid information, resulting in low training efficiency and model degradation. Lovász loss [29] is a metric-based measurement that focuses more on the metrics of the entire image instead of a single pixel. This means that there is no need to consider the problem of balanced sample distribution, and it works better for binary classification problems where the proportion of foreground samples is much smaller than the background.

Based on the above analysis, we propose to address the class-imbalance problem by applying the Lovász loss. The Lovász loss function is a smoothed expansion of the Jaccard loss for the Jaccard index. The expression for Jaccard loss is given in Equation (6):

(6) $$\Delta_{J}{}_{c}\left(y*,\tilde{y}\right)=1-\frac{\left|\left\{y*=c\right\}\cap \left\{\tilde{y}=c\right\}\right|}{\left|\left\{y*=c\right\}\cup \left\{\tilde{y}=c\right\}\right|}$$

where y* represents the predicted result of the network model, and $\tilde{y}$ represents the ground truth. As the Jaccard loss function is only applicable to the discrete case, a Lovász expansion of it can transform the input space from discrete to continuous, and the output value is equal to the output of the original function on the discrete domain. In this paper, the Lovász loss function is denoted as Δ*J_L_*.

In order to make the network optimization go toward the accurate direction of loss decline, and to enhance the supervision of parameter learning of the two texture enhancement modules, we performed loss function calculations based on three scales of feature maps. In addition to calculating the loss of the final prediction results and the labels, we also performed channel dimensionality reduction on F8 and calculateed Lovász loss with the downsampled labels and perform channel dimensionality reduction on U4 and calculate Lovász loss with the downsampled labels. The final loss is the weighted sum of the three components, as follows.

(7) $$L_{Total}=\lambda_{1}\Delta J_{L}\left(y*,\tilde{y}\right)+\lambda_{2}\Delta J_{L}\left(y^{D2}*,{\tilde{y}}_{U4}\right)+\lambda_{3}\Delta J_{L}\left(y^{D4}*,{\tilde{y}}_{F8}\right),$$

where ${\tilde{y}}_{U4}$ represents the result of dimensionality reduction on the output of U4, *y^D^*^2^* and *y^D^*^4^* denotes the result of downsampling the original labels by a factor of 2 and 4, ${\tilde{y}}_{F8}$ denotes the result of channel dimensionality reduction on F8, and *λ*_1_, *λ*_2_ and *λ*_3_ are the weights of the three scale loss functions.

### 2.4. Metric

For a binary-segmentation assignment, the prediction results are divided in four sets, i.e., true positive (TP), false negative (FN), false positive (FP), and true negative (TN). TP means inferring a positive sample as positive correctly, FN means inferring a positive sample as negative wrongly, FP means inferring a negative sample as positive wrongly, and TN means inferring a negative sample as negative correctly. For evaluating performances of different networks, we choose three metrics to assess the hazard source extraction result, which are overall accuracy (OA), F_1_-score, and recall, as defined in Equations (8)–(11). Here, OA is the proportion of correctly inferred samples to all samples; precision is the proportion of correctly inferred positive samples to all inferred positive samples; recall is the proportion of correctly inferred positive samples to all actual positive samples; F_1_-score is the harmonic average of precision and recall.

(8) $$OA=\frac{TP+TN}{TP+FN+TN+FP}$$

(9) $$F_{1}=2\cdot \frac{precision\cdot recall}{presicion+recall}=\frac{2TP}{2TP+FN+FP}$$

(10) $$precision=\frac{TP}{TP+FP}$$

(11) $$recall=\frac{TP}{TP+FN}$$

## 3. Experiments

### 3.1. Implementation Details

The implementation is based on Pytorch 1.8.0, and the training machine is a server equipped with Intel(R) Xeon(R) Gold 5218 CPU, GeForce RTX 2080 Ti GPU. We use SGD with a total of four images per minibatch. All models are trained for 29,000 iterations with an initial learning rate of 0.001. Weight decay of 0.0001 and momentum of 0.9 are used. The three weights of the loss function, *λ*_1_, *λ*_2_, and *λ*_3_, were set to 0.7, 0.2, and 0.1, respectively.

### 3.2. Ablation Study

#### 3.2.1. Tradeoff between Training Data Size and Encoder Complexity

In order to investigate the parameter scale of the encoder, Vgg16, ResNet50 and ResNet101 acted as the encoder of the UNet architecture, and trained and validated on the color-coated steel house dataset, respectively. The experimental results on the validation dataset are shown in Table 3. The ResNet50 and ResNet101 obtained higher overall accuracy (OA), F_1_-score and recall compared to Vgg16. ResNet50 had slightly lower OA than ResNet101, but the more important metrics, F_1_-score and recall, were higher than ResNet101. It can be concluded that the model depth of ResNet50 is more appropriate for the scale of the hazard source dataset. Vgg16 has fewer layers and cannot extract enough abstract high-level semantic features, while the ResNet101 model is too deep for this dataset and is prone to overfitting. Therefore, the encoder of TE-ResUNet proposed in this work is constructed based on ResNet50.

#### 3.2.2. The Effectiveness of Texture Enhancement Module

In order to verify whether the texture enhancement module can bring gains to the performance of hazard source extraction, comparative experiments of ResUNet, TE-ResUNet with TE1 module removed (TE-ResNet-A), TE-ResUNet with TE2 module removed (TE-ResNet-B), and TE-ResUNet were conducted on the validation set of color-coated steel house dataset. As shown in Table 4, the performance of the models with the addition of the texture enhancement module are improved, compared to the original ResUNet, which indicates that the texture enhancement module can bring benefits to hazard source extraction. In terms of the texture enhancement module settings, the texture enhancement for the shallower layers (TE1) is better than the texture enhancement for the deeper layers (TE2), and the two texture enhancement modules work together obtained the optimal performance.

**Figure 5:**
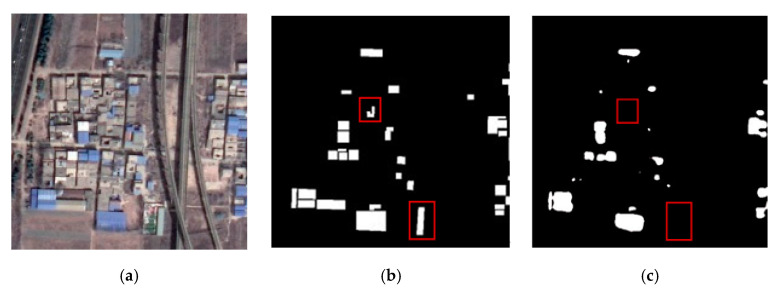

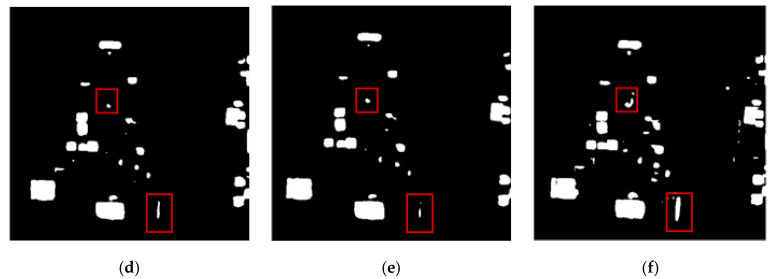
Network performance with texture enhancement module: (**a**) original image; (**b**) ground truth; (**c**) ResUNet; (**d**) TE-ResUNet-A; (**e**) TE-ResUNet-B; (**f**) TE-ResUNet.

Figure 5 shows the extraction results of each method on the validation set of color-coated steel house dataset. The extraction results of the original ResUNet (Figure 5c) show that the boundaries of the color-coated steel houses are blurred, and there are obvious omissions (see red box). The experimental results of TE-ResUNet-A (Figure 5d) and TE-ResUNet-B (Figure 5e), with the help of the texture enhancement module, have better accuracy of the boundary extraction, but still have obvious omissions. The visual effect of TE-ResUNet-B is slightly better than TE-ResUNet-A, indicating that the texture enhancement is more effective for lower-level features. As shown in Figure 5f, with the help of texture enhancement modules of two scales, TE-ResUNet has greatly improved the boundary extraction results of the color-coated steel house, and the omissions has been significantly improved.

#### 3.2.3. Network Performance with Different Loss Functions

For our dataset, the number of positive samples is significantly smaller than the negative samples. Moreover, many hazard sources are very small or with complicated and blurred boundaries, which are difficult for a network to identify. Therefore, we tested several specific loss functions to address such class imbalance problem, including the cross-entropy loss, the focal loss, the dice loss, the Lovász loss, and the proposed multi-scale Lovász loss.

**Figure 6:**
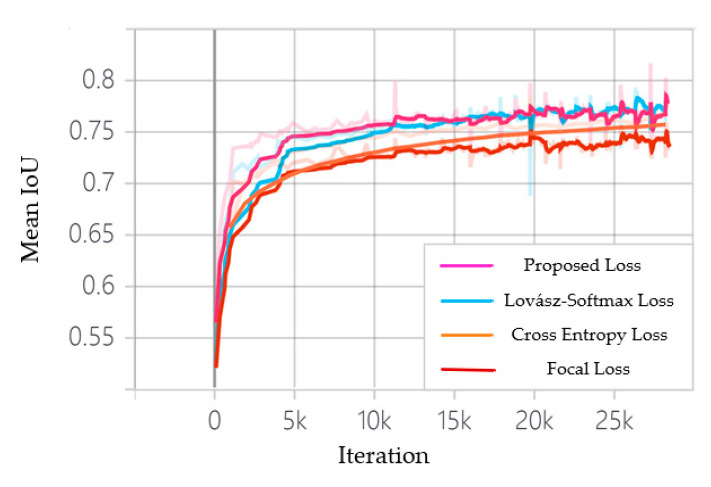
Mean IoU curves on 29 k iterations with different loss function.

As different loss functions have different rules for calculating loss values, the loss curves are not comparable, therefore the mean IoU curves of the training data are shown in Figure 6. As can be seen that the mean IoU converges to around 75% on the training data when the network is trained using pixel-based loss functions, the cross-entropy loss, and the focal loss. The curve of the cross-entropy loss is smoother and convergences to a higher mean IoU score than the focal loss. This is because the focal loss mainly solves the imbalance of the number of hard and easy samples, and has a limited effect on the imbalance of the number of positive and negative samples in this work. In contrast, the metric-based loss function, Lovász loss, and multi-scale Lovász loss outperform the pixel-based loss function, with the mean IoU converging at around 78% on the training data, demonstrate that metric-based loss function can better overcome the problem of positive and negative sample imbalance. Further, training the network with the proposed multi-scale Lovász loss results in faster convergence and a higher mean IoU score compared to the Lovász loss. This is mainly due to the multi-scale Lovász loss allows better supervision of the texture enhancement modules, which enables the network to better recover texture details and improve the accuracy of hazard source boundaries and small targets. Table 5 shows the network performance on the validation set of color-coated steel house dataset with different loss functions, further proving the superiority of the proposed loss function.

It is worth mentioning that we also conducted experiments on dice loss, which also belongs to metric-based loss, but it cannot converge on the training data. Therefore, the experimental results are not shown in Figure 6 and Table 5. The main reason may be that dice loss is more suitable for extremely imbalance samples. Under normal circumstances, using dice loss will adversely affect backpropagation, and make training unstable.

### 3.3. Comparing Network Performance with Other Method

To further validate the effectiveness of the proposed method, the experimental results on two testing sets of TE-ResUNet are compared with existing methods in this section, including FCN8s [3], PSPNet [7], DeepLabv3 [8], and AEUNet [16]. Among these methods, FCN8s is a base semantic segmentation network with 8x upsampled prediction. PSPNet extends pixel-level features to global pyramid pooling to improve the segmentation result. DeepLabv3 introduces a decoder, which can achieve accurate semantic segmentation and reduce the computational complexity. AEUNet is a spatial-channel attention-enhanced building extraction model, which introduces Resnet and attention models into UNet architecture, and proposes a multi-scale fusion module to retain the local detail characteristics. To ensure the fairness of the comparison, ResNet50 is adopted as the backbone networks of PSPNet, DeepLabv3, and AEUNet.

Table 6 shows the comparison results of each method on the color-coated steel house testing set. It can be seen that TE-ResUNet outperforms other comparison methods on all evaluation metrics. There are many small targets in the color-coated steel house dataset, so most methods have relatively serious omissions of small targets, and therefore a lower recall. While TE-ResUNet can guarantee a relatively high accuracy for small targets, and recall is improved by more than 5% compared to other methods. FCN8s has a relatively poor performance in color-coated steel house extraction due to its shallow network layers and relatively simple decoder design. The pyramid pooling module in PSPNet further downsamples the feature map on the basis of 32 times the downsampling of Resnet50, which affects the extraction accuracy of small color-coated steel houses, therefore the F_1_-score and recall are relatively low. In addition, PSPNet is difficult to train with a small dataset. Due to the atrous convolution structure of DeepLabv3, the downsampling loss of small targets is small, therefore the recall and other evaluation indicators are relatively high. AEUNet is a promising architecture with spatial and channel wise attention mechanisms to improve the representation of targets, but it requires a relatively large amount of training data to motivate its performance. The hazard source dataset is relatively small, therefore AEUNet achieves better performance in OA but lower in F_1_-score and recall when compared to the TE-ResUNet. The proposed TE-ResUNet proposed makes full use of multi-scale features through skip connections, and performs texture enhancement on low-level features to make full use of texture detail features. The texture enhancement module is lightweight and only brings very little extra cost, which can better overcome the overfitting problem caused by the insufficient data amount. Therefore, TE-ResUNet obtains more accurate boundaries and improves the recall of small objects, as well as other evaluation metrics.

**Figure 7:**
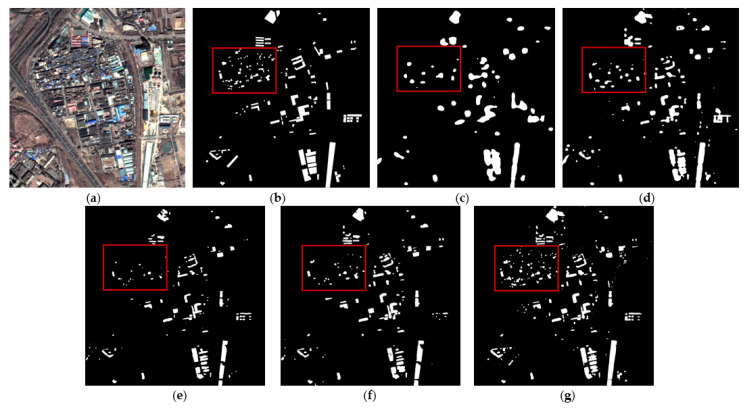
The example results of color-coated steel house testing set: (**a**) original images; (**b**) ground truths; (**c**) FCN8s; (**d**) PSPNet; (**e**) DeepLabv3; (**f**) AEUNet; (**g**) TE-ResUNets.

**Figure 8:**
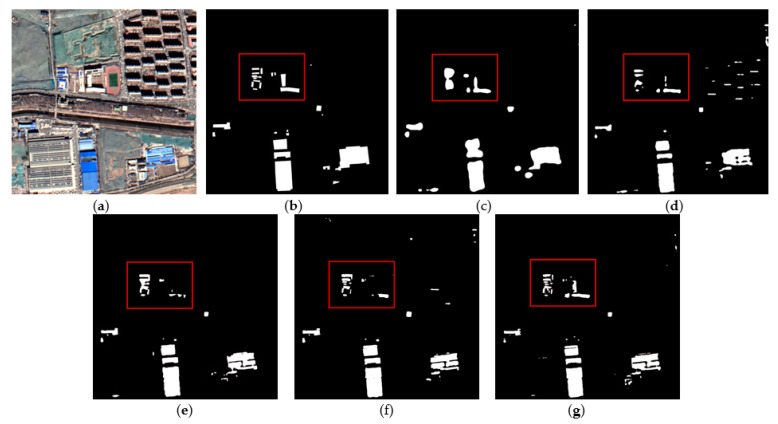
The example results of color-coated steel house testing set: (**a**) original images; (**b**) ground truths; (**c**) FCN8s; (**d**) PSPNet; (**e**) DeepLabv3; (**f**) AEUNet; (**g**) TE-ResUNets.

Figure 7 and Figure 8 show the extraction results of each method on the testing set of color-coated steel house dataset. The color-coated steel houses in the testing set are mostly adjacent to or scattered in residential areas. The spectral characteristics of the white color-coated steel houses are similar to those of the white buildings and bare soil, which are easily confused by the model. Meanwhile, there are many small targets in this dataset, which are prone to be mis-extracted by models. Figure 7c and Figure 8c are the extraction results of FCN8s. It can be seen that the extraction results of color-coated steel houses are relatively coarse. As it does not make full use of the low-level features, the boundary is blurred, and the omissions of small targets is high. Figure 7d and Figure 8d show the extraction results of PSPNet, the accuracy of the boundary is improved compared with FCN8s, but there is also the issue of omissions. Figure 7e and Figure 8e show the extraction results of DeepLabv3, the accuracy of the boundary has been greatly improved, but the problem of omissions of small targets is still serious. Figure 7f and Figure 8f show the extraction results of AEUNet, the extraction results of small targets is notably better than FCN8s, PSPNet and Deeplabv3, but the mis-extraction still exists. Figure 7g and Figure 8g show the extraction results of the proposed method, the accuracy of the boundary and the detection rate of small targets have been greatly improved (in the red box), especially for the small color-coated steel houses scattered in residential areas.

On the dust-proof net dataset, FCN8s, PSPNet, DeepLabv3, and AEUNet are also performed to compare with the proposed TE-ResUNet. Table 7 displays the experimental results of each method on the testing set of dust-proof net dataset. It can be seen that TE-ResUNet obtained the best scores for all metrics compared with other methods, which further proves the rationality of the network structure design of the proposed network. Compared to the color-coated steel houses, the dust-proof nets are generally larger in size and fewer in number. Therefore, TE-ResUNet performs well in all metrics, all of which are above 84%.

**Figure 9:**
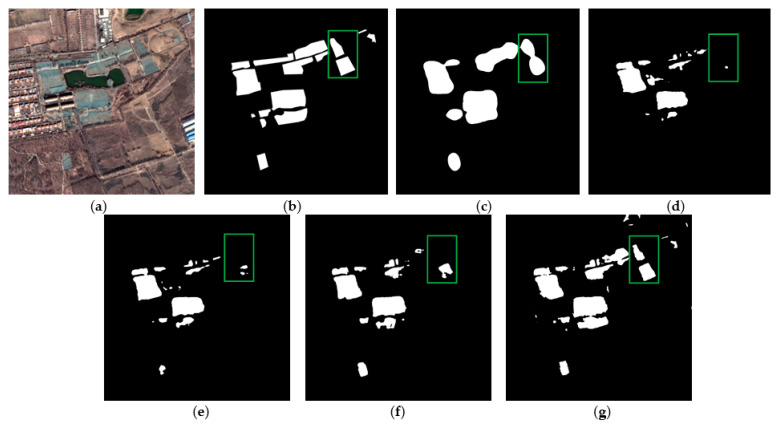
The example results of dust-proof net testing set: (**a**) original images; (**b**) ground truths; (**c**) FCN8s; (**d**) PSPNet; (**e**) DeepLabv3; (**f**) AEUNet; (**g**) TE-ResUNets.

**Figure 10:**
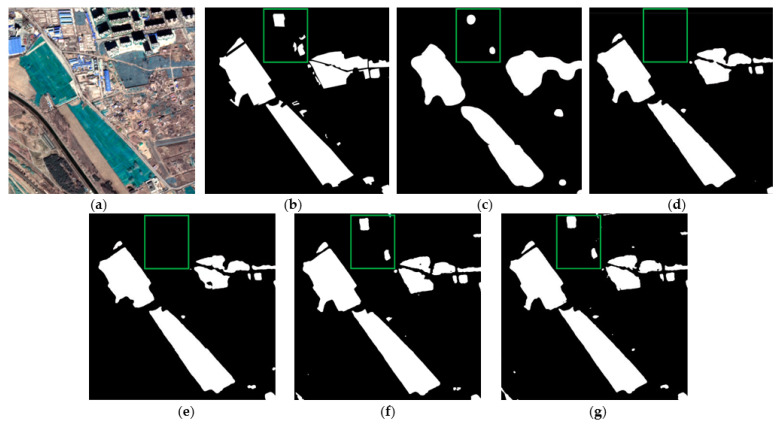
The example results of dust-proof net testing set: (**a**) original images; (**b**) ground truths; (**c**) FCN8s; (**d**) PSPNet; (**e**) DeepLabv3; (**f**) AEUNet; (**g**) TE-ResUNets.

Figure 9 and Figure 10 show the extraction results of the dust-proof net testing set. The example images contain dust-proof nets with a long coverage time, which is prone to be confused with bare soil due to fading. Figure 9c and Figure 10c show the extraction results of FCN8s—the boundary of dust-proof nets is also blurred, but the detection rate is good; Figure 9d and Figure 10d show the extraction results of PSPNet— the boundary accuracy has been improved, but the omissions are relatively high (in the green box); Figure 9e and Figure 10e show the extraction results of DeepLabv3— the boundary accuracy and omissions are improved, but omissions still occur; Figure 9f and Figure 10f show the extraction results of AEUNet—the results of dust-proof nets are more complete and fine; Figure 9g and Figure 10g show the extraction results of the proposed method—the boundary accuracy is more satisfactory and most of the dust-proof nets are extracted completely.

## 4. Conclusions

In this paper, we propose a TE-ResUNet architecture for fast-speed railway hazard source extraction from high-resolution remote sensing imagery. The TE-ResUNet is based on the UNet framework, and adopts residual connected network to deepen the model in the encoding stage. In the decoding stage, in order to make full use of the low-level texture features, we propose to introduce texture enhancement modules to enhance the texture features of the shallow layers to improve the boundary accuracy of hazard source extraction. In addition, a multi-scale Lovász loss function is proposed for model optimization, which can better cope with the sample imbalance problem, as well as perform better supervision on the texture enhancement module. The testing results on the GF-2 railway hazard extraction dataset show that the performance of the TE-ResUNet exceeds several baseline methods across all evaluation metrics.

## Figures and Tables

**Table 1:** Training samples of hazard source dataset.

Hazard Source	Number of Training Samples	
	Original Images (1000 × 1000)	Data Augmentation (512 × 512)
Color-coated steel house	153	3672
Dust-proof net	139	3336

**Table 2:** The architecture of TE-ResUNet.

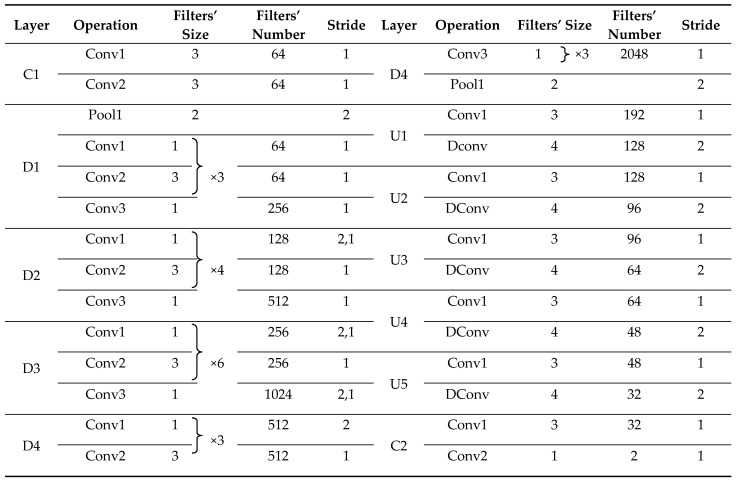

**Table 3:** Network performance with different encoders.

Encoder	OA (%)	F_1_-Score (%)	Recall (%)
Vgg16	95.32	60.22	68.30
ResNet50	95.79	64.85	72.97
ResNet101	95.98	63.78	70.25

**Table 4:** Network performance with texture enhancement module.

Models	TE1	TE2	OA (%)	F_1_-Score (%)	Recall (%)
ResUNet	×	×	95.79	64.85	72.97
TE-ResUNet-A	×	√	96.22	66.13	73.12
TE-ResUNet-B	√	×	96.83	71.13	77.38
TE-ResUNet	√	√	97.01	75.89	77.43

**Table 5:** Network performance with different loss functions.

Loss Function	OA (%)	F_1_-Score (%)	Recall (%)
Cross-entropy Loss	95.91	72.44	75.68
Focal Loss	95.32	70.32	75.00
Lovász Loss	97.01	75.89	77.43
Multi-Scale Lovász Loss	96.93	76.21	77.76

**Table 6:** Network performance on color-coated steel house testing set.

Models	OA (%)	F_1_-Score (%)	Recall (%)
FCN8s	95.95	65.62	68.15
PSPNet	97.08	70.32	60.30
DeepLabv3	96.76	72.06	73.68
AEUNet	96.51	74.93	76.58
TE-ResUNet	97.49	77.55	78.64

**Table 7:** Network performance on dust-proof net testing set.

Models	OA (%)	F_1_-Score (%)	Recall (%)
FCN8s	95.04	83.26	83.64
PSPNet	95.30	82.57	71.97
DeepLabv3	95.89	86.44	80.69
AEUNet	97.60	83.35	82.31
TE-ResUNet	96.62	88.10	84.52

## Data Availability

Data sharing not applicable.
